# Cervical Hyperostosis Leading to Dyspnea, Aspiration and Dysphagia: Strategies to Improve Patient Management

**DOI:** 10.3389/fsurg.2018.00033

**Published:** 2018-04-24

**Authors:** Georgios Psychogios, Monika Jering, Johannes Zenk

**Affiliations:** Department of Otorhinolaryngology, Head and Neck Surgery, Klinikum Augsburg, Augsburg, Germany

**Keywords:** cervical hyperostosis, dyspnea, dysphagia, aspiration, forestier’s disease

## Abstract

Diffuse idiopathic skeletal hyperostosis (DISH) is a rare but well known cause of dysphagia. In very few cases aspiration and dyspnea are described as a clinical manifestation. An 82-year-old man presented himself in our clinic with severe dyspnea, aspiration, and pneumonia. After performing a microlaryngoscopy an emergency tracheotomy became necessary.

In laryngoscopy a severe bulging of the posterior oropharyngeal and hypopharyngeal wall was detected. The glottis area was not observable and immobilisation of the right vocal cord could be detected. The CT showed anterior osteophytes and ossification of the anterior longitudinal ligament from C2–C7. We performed a panendoscopy in order to explore the upper aerodigestive area. Postoperatively an emergency tracheotomy was needed due to the development of laryngeal edema. The osteophytes were removed in cooperation with the department of orthopaedics. Three months postoperative the patient had no dyspnea or dysphagia, so the tracheotomy could be closed.

Cervical hyperostosis is commonly described in elderly patients and usually presenting without symptoms, therefore a surgical treatment is usually not necessary. Nevertheless it can lead to severe morbidity and dyspnea with airway obstruction. Therefore it is essential that cervical hyperostosis is recognized early enough and appropriate treatment is initiated. Flexible endoscopy should be preferred over direct panendoscopy because it could lead to life-threatening edema and a prophylactic tracheostomy should be strongly considered in patients that present with severe dyspnea,

## Introduction

Diffuse idiopathic skeletal hyperostosis (DISH) also known as cervical hyperostosis, ankylosing hyperostosis or Forestier’s disease is usually found in the elderly with an estimated incidence of 12–30% in men over 65 years (1–3). It was first described by Forestier and Rotes-Querol in 1950 (4) and usually presents as an asymptomatic ossification of the paraspinal connective tissue, the peripheral portion of the annular disc and the anterior longitudinal ligament (5). Clinical manifestation is occasionally described and generally includes spinal stiffness, neck-shoulder pain, globus sensation, dysphagia or non-specific symptoms such as weight loss. Complications such as intubation difficulties, dysphonia, aspiration pneumonia, stridor or dyspnea are extremely rare but can be life threatening. The need of emergency tracheotomy in patients with Forestier’s disease is only described 4 times in the literature (5–8). Identifying patients with DISH as a potential cause for unexplained symptoms is essential, especially when life-threatening airway obstruction is existent (2). The laryngeal anatomy is often changed due to osteophytes, with secondary inflammation of the mucosa and soft tissue (2).

The diagnosis is primarily radiological and relies on following definitions: (1) flowing calcification and ossification within the anterior longitudinal ligament involving four or more contiguous vertebral bodies, (2) minimal to no degenerative disc changes and (3) absence of apophyseal joint ankylosis and sacroiliac erosion (9). The last criterion was made to distinguish DISH from degenerative spondylosis. Today DISH is considered to be an extreme variant of degenerative spondylosis in some patient therefore the last criterion can be neglected (10).

We describe a case with a patient suffering of acute dyspnea and aspiration pneumonia. After undergoing a microlaryngoscopy an emergency tracheotomy was necessary due to laryngeal oedema. Strategies on how to improve patient management and an overview of the current literature is given in this case report.

## Case Report

An 82-year-old man presented in our emergency department with dyspnea, inspiratory stridor and fever. He was treated without success for a tracheo-bronchitis for about two weeks in the department of internal medicine. During admission, he confirmed 20 kg weight loss over the past 3 months, associated with a progressive difficulty in swallowing accompanied by foreign body sensation. Past medical history was remarkable for hypertension, benign prostate hyperplasia, smoking and alcohol abuse. A Dupuytren’s contracture of the left hand had been surgically released in 1970.

**Figure 1:**
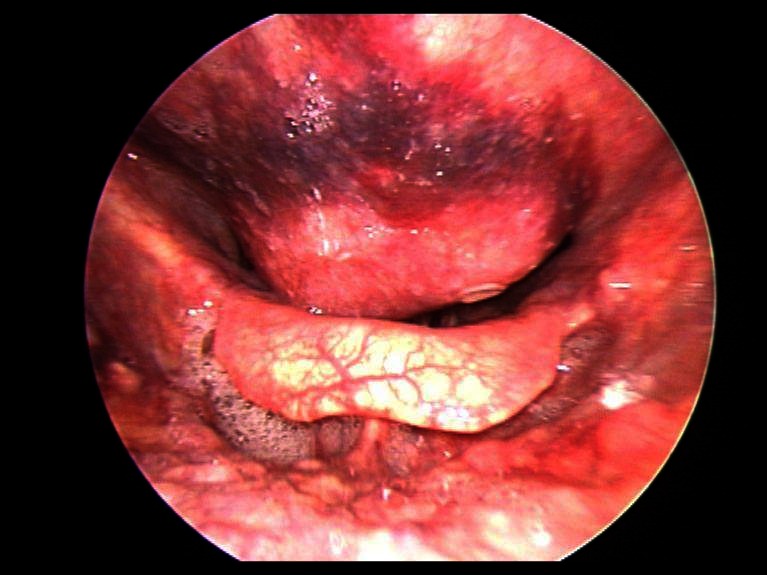
Laryngoscopic findings preoperatively.

 Microlaryngoscopy revealed protrusion of the posterior oropharyngeal and hypopharyngeal wall, which left a thin cleft to the epiglottis (Figure 1). The pharyngeal mucosa was thickened. The glottis area could not be overseen but immobilisation of the right vocal cord was discovered.

**Figure 2:**
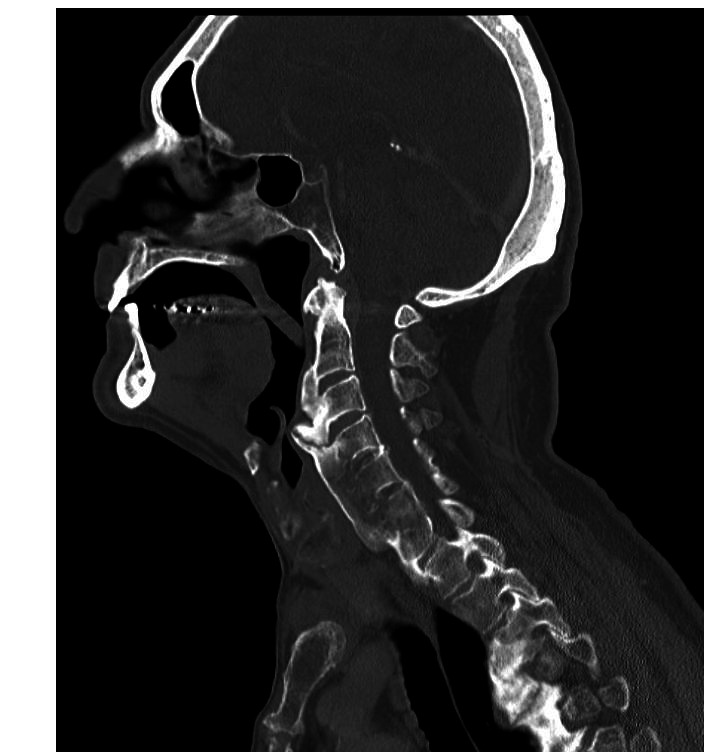
CT: sagittal scan showing anterior osteophytes and ossification of the anterior longitudinal ligament.

**Figure 3:**
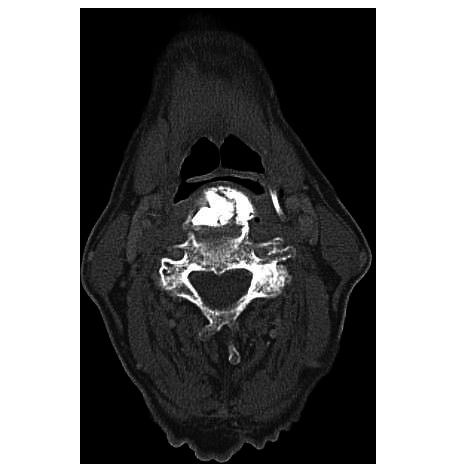
CT: axial scans showing a thin cleft between epiglottis and anterior osteophytes at the level of C3.

The preoperative CT of the C-spine and thorax showed large anterior osteophytes and ossification of the anterior longitudinal ligament from C2–C7, with a maximum at the level of C3–C4. Morphological imaging indicated the diagnosis of DISH (Figures 2 and 3). Contrast agent swallow demonstrated massive aspiration.

After initial treatment with antibiotics and corticosteroids, a panendoscopy was performed to exclude malignancy and to place simultaneously a percutaneous gastrostomy to secure the nutrition. Intraoperatively normal action potentials of the vocal cord were measured by using electromyography. Postoperatively the patient developed laryngeal edema necessitating emergency tracheotomy.

**Figure 4:**
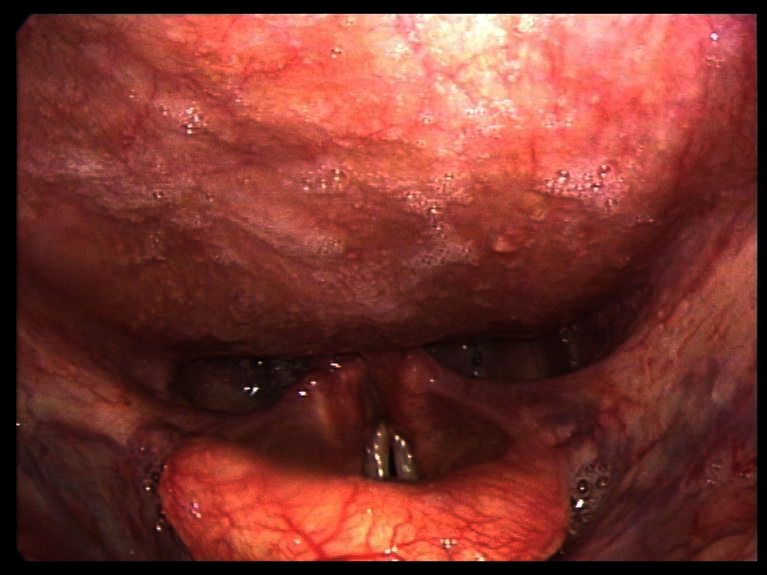
laryngoscopic findings 3 months postoperatively.

In the department of orthopaedics osteophytes were excided through an anterior lateral extrapharyngeal approach. After three months the patient had no breathing problems and no aspiration, so the tracheotomy could be closed (Figure 4). Unfortunately the vocal cord paralysis of the right side and the hoarseness of the patient did persist. Written informed consent was obtained from the patient described in this case report.

## Discussion

Cervical hyperostosis or Forestier’s disease is mostly an asymptomatic malady described as a noninflammatory ossification with severe formation of osteophytes affecting ligaments, tendons, and fasciae, particularly of the spinal column (11). Why this entity usually remains asymptomatic and which patient group become symptomatic remains unknown.

Resnick et al. noted that the syndrome was a more systemic process, and diffuse idiopathic skeletal hyperostosis (DISH) was suggested as a more appropriate description of this ossifying diathesis (12). An otolaryngologist is consulted if a patient presents with dysphagia, aphagia, and globus sensation. Typically a patient undergoes many different diagnostic procedures before DISH is diagnosed (7).

The Otolaryngologist will perform an endoscopic laryngoscopy and will find a submucosal protrusion of the posterior wall of the pharynx. To confirm the diagnosis barium esophagogram and CT scans are necessary. Most commonly involved are level C4 and C5, although other or multiple levels can be affected. The cricoid is usually located between C4 and C6, where the oesophagus is typically attached, therefore most patients suffer from dysphagia (7). Involvement of the level C2 and C3 will not cause dysphagia but can cause globus sensation, stridor and respiratory distress if the osteophytes become very large.

Known risk factor for DISH include older age and diabetes (13). A correlation is discussed between DISH and excessive weight, hyperuricemia and Dupuytren’s contracture in men over the age of 60.

DISH can rarely cause dysphagia. Oropharyngeal dysphagia in the elderly is usually caused either by neuromuscular disorders such as stroke, Parkinson disease, multiple sclerosis or myasthenia gravis or by structural disorders (14). A variety of lesions such as tumor, inflammatory processes and Zenker's diverticulum may cause oropharyngeal dysphagia due to obstruction (15). If the main symptom is serious obstruction the method of choice is operative resection of the osteophytes without fusion (6). Surgical treatment was described as being a successful method to relief symptoms. A fusion after osteophytectomy for DISH presenting with dysphagia is not needed as described in several published case reports (16).

Respiratory related problems in patients with DISH are very rare but can be life-threatening (17). Most of the cases with airway compression are associated with cervical osteophytes at level C2 and C3, as the posterior pharyngeal wall and larynx can be restricted. Occasionally dyspnoea is the main symptom, however a mechanical obstruction is not necessarily visible (7). In our case dyspnoea was caused by obstructing osteophytes and a reactive inflammatory hyperplasia, and vocal cord immobilisation. In cases like that, direct panendoscopy should be avoided if possible, because surgical trauma can lead to life-threatening edema. A carefully performed flexible endoscopy usually is enough and can help avoid complications. On the other hand in cases that present with severe airway obstruction, surgical ostreophyte removal can also lead to postoperative dyspnoea and prophylactic tracheotomy should be considered (1). Awaken fiberoptic intubation is the standard procedure while managing the airway of DISH patients, as major complications include intubation difficulty and spinal cord injury.

Cervical osteophytes may also cause dyspnoea due to bilateral vocal cord paralysis or obstruction, and therefore DISH is a differential diagnosis to vocal cord paralysis (18). Furthermore a chondritis could occur with subsequent involvement of the arytenoids and ankylosis of the cricoarytenoid joint, which is likely to be the reason why the vocal cord immobilisation of our patient did not improve (8). Another possible reason could be an infection of the recurrent nerve itself (10, 19). We could exclude this in our patient because the intraoperative electromyography of the vocal muscle on both sides showed normal action potentials (20).

Airway obstruction as a complication of DISH has to be treated with operative excision of osteophytes if conservative therapy fails (13, 21). The most common operative technique is an anterior-lateral extrapharyngeal approach. Postoperative complications include vocal cord paralysis and perforation of the pharynx or oesophagus (22).

In conclusion Forestier’ disease is a common finding in the elder patients and normally asymptomatic, thus a surgical treatment is not required (23). The most common symptom seen by the otolaryngologist is dysphagia. Dysphagia in combination with dyspnoea can be potentially life-threatening, therefore a prompt diagnosis and adequate treatment is necessary. Flexible endoscopy should be the preferred method of examination in order to avoid complications and imaging with CAT scan will most frequently obviate the need for direct panendoscopy. If the latter is deemed necessary after all, it should be considered very cautiously because, as illustrated in our report, it can lead to life-threatening edema. In patients that present with severe dyspnoea, a prophylactic tracheostomy should be strongly considered. In retrospect, our patient would have profited from this treatment strategy.

## Author Contributions

GP: treatment of patient, text writing, literature and figures. MJ: text writing and literature. JZ: treatment of patient and text writing.

## Conflict of Interest Statement

The authors declare that the research was conducted in the absence of any commercial or financial relationships that could be construed as a potential conflict of interest.
